# *C. perfringens* challenge reduces matrix metalloproteinase activity in the jejunal mucosa of *Eimeria*-infected broiler chickens

**DOI:** 10.1186/s13567-020-00825-6

**Published:** 2020-08-08

**Authors:** Lore Van Damme, Natasja Cox, Chana Callens, Freddy Haesebrouck, Michelle Dargatz, Richard Ducatelle, Filip Van Immerseel, Evy Goossens

**Affiliations:** 1 Department of Pathology, Bacteriology and Avian Diseases, Faculty of Veterinary Medicine, Ghent University, Merelbeke, Belgium; 2 Division Nutrition & Care-Animal Nutrition, Evonik Operations GmbH, Halle, Westfalen 33790 Germany

**Keywords:** broiler, *Clostridium perfringens*, *Eimeria*, gut, matrix metalloproteinases, necrotic enteritis

## Abstract

Matrix metalloproteinases (MMPs) play an important role in intestinal extracellular matrix homeostasis. An overexpression of MMPs results in tissue destruction and local inflammation and has been associated with multiple inflammatory diseases. These host proteases might also be important in tissue damage caused by infectious agents, such as in intestinal damage in *Clostridium perfringens*-induced avian necrotic enteritis (NE). The aim of the present study was to elucidate the effect of a *C.* *perfringens* infection on the MMP activity in the small intestine of birds with a pre-disposing coccidial infection to obtain a more thorough understanding of the pathogenesis of NE. For this purpose, the gelatinolytic activity present in jejunal tissue of *Eimeria* infected birds which were challenged with either a pathogenic *C. perfringens* type G strain or a commensal *C. perfringens* type A strain was analyzed using substrate zymography. The results show that infection of broilers with *Eimeria* and different *C.* *perfringens* strains, independent of their pathogenicity, decreases the expression of a 40–45 kDa host collagenase in the jejunum, as compared to the expression in *Eimeria*-infected control birds. It was also shown that the expression of 2 MMPs with molecular weights of approximately 50–60 and 60–70 kDa was significantly lower in necrotic tissue as compared to the activity in macroscopically healthy tissue adjacent to the lesion. These results indicate that host collagenases are not elicited by the *C. perfringens* infection for permeabilizing the host mucosa to allow penetration of the NetB toxin in *Eimeria* infected broilers.

## Introduction

Necrotic Enteritis (NE) is one of the most common and financially devastating bacterial diseases in the modern poultry industry [1, 2]. The disease is caused by *netB*-positive *C. perfringens* strains and is characterized by severe necrosis and inflammation of the small intestine [3–5]. The pore-forming toxin NetB is an essential virulence factor in the development of necrotic ulcers which are typical for NE in broilers [4]. Where and how NetB initiates damage is hitherto not determined. It is currently believed that NetB is not involved in early disease pathogenesis and targets deeper layers of the intestinal mucosa rather than superficial structures [6]. Therefore, initial breakdown or permeabilization of the mucosal layer might be required in order for NetB to exert its action. This permeabilization can be either a direct effect on the host tissues of *C.* *perfringens* virulence factors other than NetB, or it can be a host response elicited indirectly by *C.* *perfringens*.

Overexpression of host collagenases has been associated with intestinal tissue destruction in several gastro-intestinal inflammatory diseases in humans, including Crohn’s disease, ulcerative colitis and several forms of gastro-intestinal cancer [7–11]. These host collagenases might also be important in tissue damage caused by infectious agents, such as in intestinal damage in NE. Indeed, collagen is widely distributed throughout the gastrointestinal tract and is an integral component of the connective tissue and basement membrane of the intestinal mucosal layer [12–14]. Disruption of this structural protein may result in loss of tissue integrity and allow penetration of bacterial toxins to deeper tissues and so contribute to subsequent tissue necrosis [15]. Furthermore, Olkowski and colleagues showed an increased MMP activity in necrotic tissue of broilers which were challenged with NE-producing *C. perfringens* strains as compared to tissue of non-challenged birds [16].

NE is a multifactorial disease and outbreaks of NE are almost exclusively found to co-occur or follow upon a predisposing coccidial infection [17–21]. During coccidiosis, *Eimeria* spp. colonize the intestine and destroy epithelial cells as a consequence of the intracellular stages of their lifecycle [20]. *Eimeria* induces leakage of serum proteins in the lumen of the gut and increases mucus secretion. Both mucus and serum proteins are rich sources of nutrients which *C. perfringens* can exploit for proliferation and toxin production [22, 23]. In addition to providing nutrients for *C. perfringens*, a predisposing *Eimeria* infection also has tremendous effects on the intestinal tissue itself by shortening of the villi [24], inducing inflammation [25], reducing the activity of digestive enzymes and disrupting tissue integrity [20]. In order to fully characterize the events that contribute to disease pathogenesis, even under experimental settings, this predisposing coccidial infection should be taken into account.

Therefore, the aim of the present study was to focus on the host response to *C. perfringens*, trying to elucidate the effect of a *C. perfringens* infection on the host MMP activity in the small intestine of birds in the presence of a coccidial infection, known to be a crucial predisposing factor for NE.

## Materials and methods

### Bacterial strains and culture conditions

Two different *C. perfringens* strains were used. Strain 56 was originally isolated from the gut of a NE-affected broiler and is characterized as a toxinotype G strain. The strain is able to express NetB toxin and is routinely used to induce NE in an in vivo infection model in broilers [26]. Strain JIR4857 is characterized as a commensal toxinotype A strain and cannot express NetB toxin. The inoculum for the oral infection of chickens was prepared by culturing both strains anaerobically overnight at 37 °C in brain heart infusion broth (Oxoid, Basingstoke, UK).

### Necrotic enteritis trial

The in vivo NE model used in this trial was based on a previously described study [27]. In short, 1-day-old unvaccinated Ross 308 broilers were randomly allocated to 3 different treatment groups with 27 birds/pen (4 replicate pens challenged with strain CP56, 1 pen challenged with the commensal *C. perfringens* strain JIR4857 and 1 pen with control birds, which were not challenged with *C. perfringens*). All broilers were fed a wheat/rye-based (43%/7.5%) diet supplemented with soybean meal as a protein source. From day 17 on, the diet was altered with fishmeal (30%) replacing the soy bean meal as a protein source. These diets contain high levels of proteins and non-starch polysaccharides which predispose chicken to the development of NE. Mild immunosuppression was induced by administering the commercial Nobilis Gumboro D78 vaccine, containing attenuated infectious bursal disease virus, on days 4 and 9 (MSD Animal Health). On days 14 and 16, all animals received a tenfold dose of live attenuated *Eimeria* vaccines, respectively Hipracox (containing 5 *Eimeria* species: *E. tenella, E. acervulina, E. maxima, E.* *praecox and E. mitis*) (Hipra, Melle, Belgium) and Paracox-8 (containing 7 *Eimeria* species: *E.* *acervulina, E.brunetti, E. maxima, E. mitis, E. necatrix, E. praecox* and *E. tenella*) (MSD Animal Health, Brussels, Belgium), to induce a predisposing coccidial infection. On days 18, 19 and 20, birds in the first group were challenged with approximately 5 × 10^8^ CFU of *netB*-positive *C. perfringens* strain 56. Birds in the second and third group received the same predisposing factors as the first group but were inoculated on day 18, 19 and 20 with respectively 5 × 10^8^ CFU of *netB*-negative *C. perfringens* strain JIR4857 or sterile bacterial growth medium. On day 21, all animals were euthanized.

At necropsy, NE severity was evaluated by scoring lesions in the small intestine (duodenum, jejunum, ileum) as previously described by Keyburn et al. [28] as follows: score 0 = no lesions, score 2 = focal necrosis or ulcerations (1–5 foci), score 3 = focal necrosis or ulcerations (6–15 foci), score 4 = focal necrosis or ulcerations (≥ 16 foci), score 5 = patches of necrosis of 2–3 cm long, score 6 = diffuse necrosis. Birds with a lesion score of 2 or more were considered NE positive. From each scoring class, jejunal lesion tissue (except for scoring class 0) and macroscopically unaffected tissue, 1 cm adjacent to the lesion of 5 different animals was collected. The samples that were collected from NE-affected birds were derived from 2 different pens (5 samples were collected from pen 1, 4 samples were collected from pen 2). The samples were stored at − 20 °C.

### Protein extraction

Proteins were extracted from the intestinal tissue using mechanical lysis. Briefly, ± 27 mg of intestinal tissue was mixed with 400 µL TBS-1% NP-40 [50 mM Tris/HCl, pH 8.0, 150 mM NaCl and 1% (v/v) NP-40 supplemented with EDTA-free protease inhibitor cocktail (Complete, Roche, Mannheim, Germany)]. The mixture was homogenized by grinding with 2.3 mm zircon/silica and 3.2 mm stainless steel beads (BioSpec Products, Bartlesville, OK, USA) in a bead beater (twice for 1.5 min, 22.5 Hz; TissueLyser) with a 30 s interval between shakings. Subsequently, samples were centrifuged for 10 min at 8000 × *g* and the supernatant was transferred to a new tube. Protein concentration was measured using the BCA protein assay (Thermo Fisher Scientific, Merelbeke, Belgium) and samples were stored at − 20 °C until further analysis.

### Zymography

The gelatinolytic activity in the intestinal tissue lysates collected from the NE trial was analyzed using gelatin zymography. Briefly, aliquots containing 60 µg of protein of each sample were mixed with 2× loading buffer (0.5 M Tris–HCl pH 6.8, 20% glycerol, 4% SDS, a pinch of bromophenol blue) and separated on 8% SDS page containing 0.1% gelatin under non-denaturing conditions. After separation, the gel was incubated with renaturing buffer (2.5% Triton X-100, 30 min, room temperature) to remove SDS from the gel. This allows the separated enzymes in the gel to renature and auto-activate. Subsequently, the gel was washed with developing buffer (150 mM NaCl, 5 mM CaCl_2_, 0.05% NaN3 and 50 mM Tris–HCl buffer pH 7.5) and incubated with fresh developing buffer under continuous shaking at 37 °C for 18 h. Afterwards, the gel was stained for 1 h with Coomassie Brilliant Blue (Sigma-Aldrich, Overijse, Belgium) and destained for 20 min with destaining solution [40% methanol (v/v), 10% acetic acid (v/v)]. Activity of gelatin-degrading enzymes is visible as clear colorless bands against a blue background. Gels were scanned using a GS-800 calibrated densitometer and the approximate molecular weight of the present bands and their intensities were determined using the Quantity One software (BioRad, Hercules, CA, USA). The gelatinolytic activity of each band was calculated as $\frac{100}{{OD/{\text{mm}}^{2} }}$ and is described in arbitrary units (AU). Six different gelatinolytic bands were observed in the jejunal tissue. The lowest and highest bands were not quantified due to distortion and oversaturation of the bands.

### Statistical analysis

Differences in the occurrence of NE lesions between the different segments of the small intestine of *C. perfringens* type G challenged broilers were evaluated by a binary logistic regression analysis using R statistical software. All other analyses and calculations were performed using GraphPad Prism software (version 5.03, San Diego, CA, USA). A paired Student’s *t* test was used to compare gelatinase activity in necrotic and macroscopically healthy tissue derived from the same bird. For all other data, two-group experiments were analyzed with unpaired Student’s t test, whereas one-way ANOVA followed by Tukey’s test was used for comparison of more than two groups. Results are presented as mean ± SEM. Analyses were performed with 95% confidence intervals and significance was determined as p ≤ 0.05.

## Results

### Lesions of necrotic enteritis are more prevalent and severe in the jejunum

Broilers of the control group which were not challenged with *C. perfringens* but received all predisposing factors including *Eimeria* infection did not develop NE (n = 26). Additionally, no necrotic lesions were seen in the intestine of birds challenged with a commensal *C. perfringens* type A strain JIR4857 (n = 27). Only *C. perfringens* type G strain CP56 was able to induce necrotic lesions in *Eimeria* infected birds. Lesions were observed in 85.05% (91/107) of the birds challenged with this *netB*–positive *C. perfringens* strain. In NE positive chickens the lesions could be observed in all segments of the small intestine, but were most severe in the jejunum as compared to the duodenum or ileum (p < 0.0001). A significant association was observed between the small intestinal segment and the development of necrotic lesions, with the jejunum being 17.89 to 22.9 times more likely to develop necrotic lesions as compared to the duodenum or ileum, respectively. Moderate enteric lesions (score 3 and 4) were dominant, being present in 68.13% (62/91) of the affected birds. Lesion score distributions for each segment of the small intestine are summarized in Table 1.

**Table 1:** **Score distribution of necrotic enteritis lesions in the small intestine of 21-day old broilers**

Group	Segment	Lesion score^a^						NE positive^b^	Average lesion score^c^
		0	2	3	4	5	6		
Control	Duodenum	26	0	0	0	0	0	0% (0/26)	–
	Jejunum	26	0	0	0	0	0	0% (0/26)	–
	Ileum	26	0	0	0	0	0	0% (0/26)	–
	Overall							0% (0/26)	–
Type A (JIR 4857)	Duodenum	27	0	0	0	0	0	0% (0/27)	–
	Jejunum	27	0	0	0	0	0	0% (0/27)	–
	Ileum	27	0	0	0	0	0	0% (0/27)	–
	Overall							0% (0/27)	–
Type G (CP56)	Duodenum	85	11	6	4	1	0	20.56% (22/107)^A^	2.77^C^
	Jejunum	19	7	15	46	18	2	82.24% (88/107)^B^	3.92^D^
	Ileum	89	6	5	6	0	1	16.82% (18/107)^A^	3.17^C^
	Overall							85.05% (91/107)	3.91

All birds were challenged with a ten-fold dose of a live attenuated Eimeria vaccine at day 14 and 16 to induce a predisposing coccidiosis infection. On day 18, 19 and 20, birds were challenged with either a pathogenic *C. perfringens* type G strains (CP56), a commensal *C. perfringens* type A strain (JIR4857) or sterile bacterial culture medium (control).

^a^NE lesion scoring of the small intestine was performed as previously described by Keyburn et al. [28].

^b^NE positive = lesion score ≥ 2.

^c^Average lesion score of NE positive birds. Data represents mean ± SE.

^d^Means within the same column with different superscripts differ significantly (*p *< 0.05); ^A, B^ binary logistic regression analysis or ^C, D^ one-way ANOVA followed by Tukey’s multiple comparisons test.

### *Clostridium perfringens* challenge decreases the activity of a specific host collagenase

As the NE lesions occurred most frequently in the jejunum of broilers, we focused on this section of the small intestine to study the effect of *C. perfringens* challenge on host MMP activity in *Eimeria* infected birds. The gelatinolytic activity present in jejunal tissue from broilers in the different treatment groups of the NE trial was assessed by gelatin zymography.

As NE is a complex disease, not all animals are equally affected by NE in experimental trials and field outbreaks. In the current trial, a minority of the birds challenged with the pathogenic, *C. perfringens* type G strain did not develop macroscopic lesions of NE. When focusing on these birds that did not develop NE (i.e. birds without macroscopic intestinal necrosis), *s*ignificantly lower gelatinase activity of a 40–45 kDa protein was measured in the jejunal tissue of birds challenged with a *C. perfringens* type A or type G strain as compared to control chickens receiving only the predisposing *Eimeria* infection (p-value respectively 0.0317 and 0.0098, Table 2). No difference in gelatin-degrading activity of this 40–45 kDa protein was observed between birds challenged with either the *C. perfringens* type A or type G strain. Furthermore, *C. perfringens* challenge of *Eimeria* infected birds did not affect the activity of the other gelatinolytic enzymes in the jejunal tissue (Table 2).

**Table 2:** **Gelatinolytic activity of jejunal samples of*Eimeria*and*C. perfringens*type A- or type** **G-infected broilers**

Group	NE	Gelatinolytic enzyme, approximate MW			
		~ 40–45 kDa	~ 50–60 kDa	~ 60–70 kDa	~ 75–85 kDa
Control (n = 5)	No	1.10 ± 0.054^A^	1.19 ± 0.072	1.03 ± 0.039	1.09 ± 0.077
Type A (n = 5)	No	0.81 ± 0.083^B^	1.10 ± 0.15	1.05 ± 0.14	1.09 ± 0.18
Type G (n = 5)	No	0.74 ± 0.051^B^	1.01 ± 0.086	0.93 ± 0.063	0.89 ± 0.087
Type G (n = 9)	Yes	0.65 ± 0.030	0.98 ± 0.045	0.86 ± 0.051	0.74 ± 0.045

Control: *Eimeria* infected birds, Type A and Type G: *Eimeria* infected birds challenged with respectively a commensal *C. perfringens* type A strain (JIR4857) or pathogenic *C. perfringens* type G strain (CP56). NE: birds without (No) or with (Yes) necrotic enteritis. Gelatinolytic activity of the digested spots is expressed in arbitrary units = $\frac{100}{{OD/{\text{mm}}^{2} }}$. Data represents mean ± SE. One-way ANOVA followed by Tukey’s multiple comparisons test was used to compare the means of the NE = No groups. Values within the same column with different superscripts differ significantly (*p *< 0.05).

### MMP activity is decreased within NE lesions

In order to assess whether the difference in disease susceptibility between birds within the *C. perfringens* type G strain challenged group might be related to an altered reaction of host MMP activity on the *C. perfringens* challenge, the gelatinolytic activity of macroscopically healthy jejunal tissue of birds that developed NE was compared to birds that did not develop NE, but were infected with the same *C. perfringens* type G strain. No differences in MMP activities were observed in macroscopically unaffected jejunal tissue of birds suffering from NE as compared to unaffected birds (Table 2).

**Figure 1:**
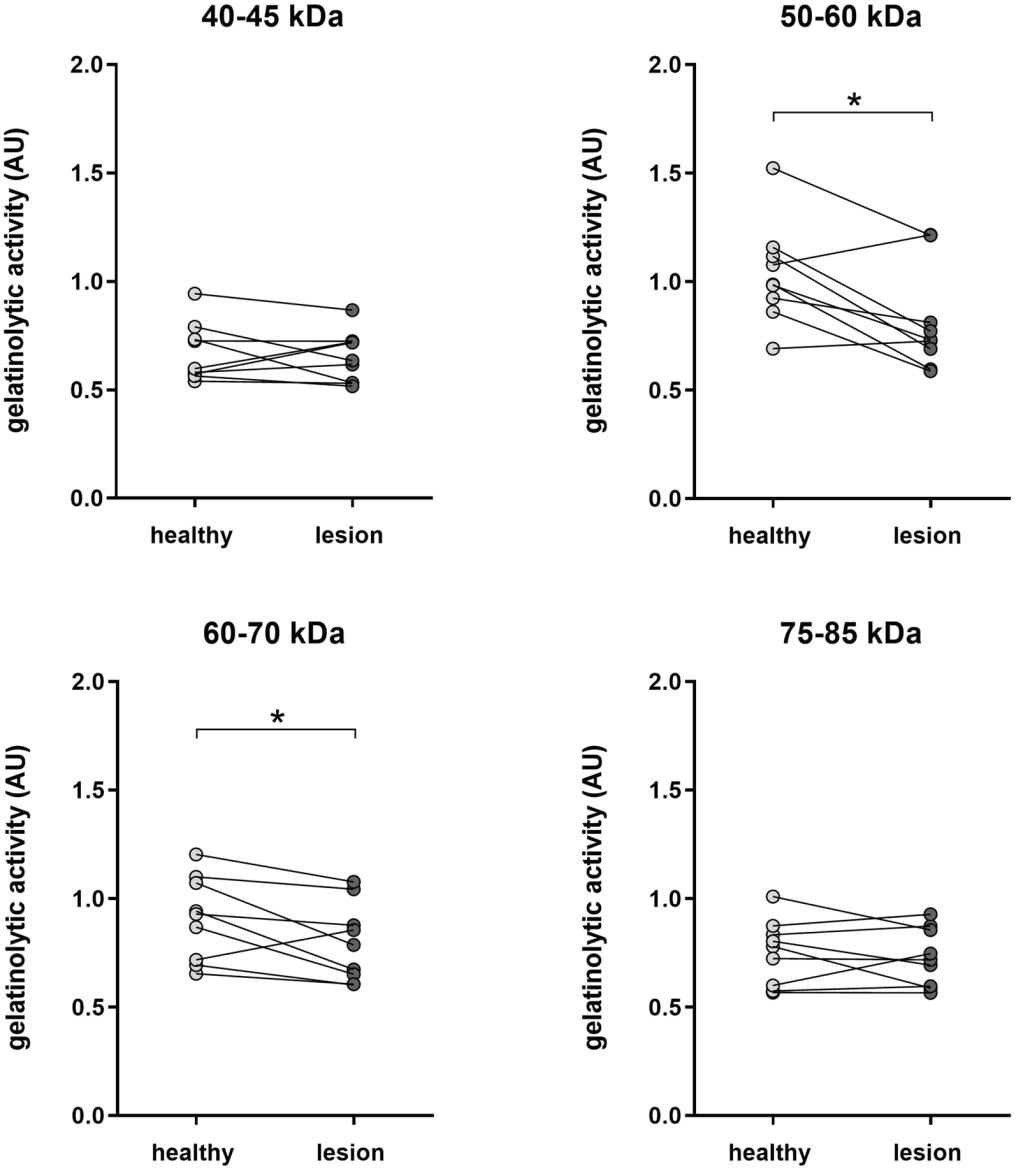
**Gelatinolytic activity in jejunal tissue of necrotic enteritis affected birds.** Lesions and macroscopically unaffected jejunal tissue 1 cm adjacent to the lesion was derived from broilers receiving a predisposing *Eimeria* infection, followed by a pathogenic *C. perfringens* type G strain (CP56) challenged. Gelatinolytic activity was analyzed by gelatin zymography and quantification of the digested spots is expressed in arbitrary units = $\frac{100}{{OD/{\text{mm}}^{2} }}$. = p ≤ 0.05 (Paired Student’s t-test).

Additionally, to assess the involvement of host MMPs in lesion development, the MMP activity within the necrotic lesions was compared to the activity in macroscopically unaffected tissue adjacent to the lesions. The activity of two MMPs with a molecular weight of approximately 50–60 and 60–70 kDa (presumably MMP2 which has a MW of ~ 62 kDa) was significantly lower in tissue derived from the lesions as compared to macroscopically unaffected tissue adjacent to the lesions (respectively p = 0.0107 and 0.0341). No difference in activity of the 40–45 kDa or 75–85 kDa enzymes was observed (Figure 1).

## Discussion

MMPs play an important role in degrading components of the extracellular matrix and basement membrane [29]. In addition to ECM degradation, MMPs are involved in multiple physiological processes, like innate and adaptive immunity and inflammation, by regulating the release and activation of cytokines, growth factors, antibiotic peptides and other bioactive molecules [30, 31]. Expression of MMPs is tightly regulated by a complex process of enzyme synthesis, secretion, activation, and inhibition, which results in low basal levels of these enzymes in healthy tissue [32]. Still, an overexpression of host collagenases results in tissue destruction and local inflammation and has been associated with multiple inflammatory diseases and pathological processes [33]. In this study we investigated whether challenge of broiler chickens with either a pathogenic, *netB*-positive or a non-pathogenic, *netB*-negative *C.* *perfringens* strain affects the jejunal expression of host-derived MMPs using an experimental model of NE involving a predisposing *Eimeria* infection.

As expected, only the *netB*-positive *C. perfringens* type G strain was able to induce NE. Postmortem examinations of NE affected birds showed that necrotic lesions are more prevalent and severe in the jejunum compared to other segments of the small intestine. The distribution of intestinal lesions in the present infection study is in accordance with older studies of both laboratory consignments and field cases of NE [18]. Why this segment of the small intestine is more prone to develop lesions is hitherto unknown.

Clostridial challenge of *Eimeria* infected birds significantly reduced the gelatinolytic activity in the broiler jejunum as compared to unchallenged *Eimeria* infected control birds. Indeed, even when no macroscopic lesions were observed, challenge with either a non-pathogenic type A strain or a pathogenic *C.* *perfringens* type G strain both resulted in reduced activity of a specific intestinal collagenase with a molecular weight of 40-45 kDa. Moreover, the activity of two larger MMPs was significantly reduced (an unknown MMP with a MW of 50-60 kDa and a 60-70 kDa MMP, presumably MMP2) in necrotic tissue as compared to the activity in macroscopically healthy tissue adjacent to the lesion. This is in contrast to previous findings from Olkowski et al., where a significant increase of MMP activity was observed in necrotic tissue as compared to the activity in healthy tissue from unchallenged control birds [16]. However, it should be noted that no comparison with unaffected tissue from *C. perfringens* challenged birds was made. Furthermore, Olkowski et al. induced NE by two oral *C. perfringens* inoculations without a preceding *Eimeria* infection [16]. Yet, coccidiosis plays an important role in the occurrence and severity of outbreaks of NE and is commonly used as a predisposing factor in experimental NE models [17]. Therefore, in the present study, a necrotic enteritis challenge model was used that includes a predisposing *Eimeria* infection caused by administering an overdose of commercially available coccidial vaccines which is followed by the administration of *C. perfringens* for 3 consecutive days.

Park et al. already demonstrated a differential expression of various genes involved in innate immunity when broilers were co-infected with *Eimeria* and *C. perfringens* as compared to challenge with either pathogen alone, suggesting that the host inflammatory response is fundamentally different during dual infection [34]. In the current study, we assessed the effect of *C. perfringens* challenge on host MMP activity, in the presence of a predisposing *Eimeria* infection. The contradictory findings between the current study and previous results from Olkowski et al. indicate that primary infection with *Eimeria* might increase the collagenase activity, whereas subsequent *C. perfringens* might dampen this response again. However, no reports are available studying the effect of *Eimeria* infection on the collagenase activity in the small intestine. Also in the current study no conclusions can be drawn on the effect of single *Eimeria* infection on host MMP activity, as no unchallenged birds or birds infected with *C. perfringens* alone were used. Nevertheless, our study indicates the importance of using an experimental NE model which resembles the reality in animal farming as close as possible as different models can lead to unexpected contradictory results.

In conclusion, *C. perfringens* challenge reduced the MMP activity in the jejunal tissue of *Eimeria* infected broilers. These results indicate that host collagenases are not elicited by the *C. perfringens* infection for permeabilizing the host mucosa to allow penetration of the NetB toxin in *Eimeria* infected broilers. This is in contrast to the previous reported increase of MMP activity in the necrotic lesions of *C. perfringens* challenged birds in the absence of a predisposing *Eimeria* infection. Further studies are needed to fully characterize the effect of *Eimeria* challenge, as well as the combined challenge of *Eimeria* and *C. perfringens*, on specific MMP activity in the broiler intestine, as compared to uninfected healthy controls. One interesting topic for future work includes the identification of the differential expressed host MMPs that were observed in this study.

## Data Availability

All data are available on request.

## Abbreviations

NE: necrotic enteritis
AU: arbitrary units
MMP: matrix metalloproteinase
